# Genomic dissection of additive and non-additive genetic effects and genomic prediction in an open-pollinated family test of Japanese larch

**DOI:** 10.1186/s12864-023-09891-4

**Published:** 2024-01-02

**Authors:** Leiming Dong, Yunhui Xie, Yalin Zhang, Ruizhen Wang, Xiaomei Sun

**Affiliations:** 1 State Key Laboratory of Tree Genetics and Breeding, Key Laboratory of Tree Breeding and Cultivation of State Forestry and Grassland Administration, Research Institute of Forestry, Chinese Academy of Forestry, Beijing, 100091 China; 2 Key Laboratory of National Forestry and Grassland Administration on Plant Ex situ Conservation, Beijing Floriculture Engineering Technology Research Centre, Beijing Botanical Garden, Beijing, 100093 China

**Keywords:** Genomic prediction, Dominance, Epistasis, GBLUP, RKHS, Japanese larch

## Abstract

Genomic dissection of genetic effects on desirable traits and the subsequent use of genomic selection hold great promise for accelerating the rate of genetic improvement of forest tree species. In this study, a total of 661 offspring trees from 66 open-pollinated families of Japanese larch (*Larix kaempferi* (Lam.) Carrière) were sampled at a test site. The contributions of additive and non-additive effects (dominance, imprinting and epistasis) were evaluated for nine valuable traits related to growth, wood physical and chemical properties, and competitive ability using three pedigree-based and four Genomics-based Best Linear Unbiased Predictions (GBLUP) models and used to determine the genetic model. The predictive ability (PA) of two genomic prediction methods, GBLUP and Reproducing Kernel Hilbert Spaces (RKHS), was compared. The traits could be classified into two types based on different quantitative genetic architectures: for type I, including wood chemical properties and Pilodyn penetration, additive effect is the main source of variation (38.20-67.46%); for type II, including growth, competitive ability and acoustic velocity, epistasis plays a significant role (50.76-91.26%). Dominance and imprinting showed low to moderate contributions (< 36.26%). GBLUP was more suitable for traits of type I (PAs = 0.37–0.39 vs. 0.14–0.25), and RKHS was more suitable for traits of type II (PAs = 0.23–0.37 vs. 0.07–0.23). Non-additive effects make no meaningful contribution to the enhancement of PA of GBLUP method for all traits. These findings enhance our current understanding of the architecture of quantitative traits and lay the foundation for the development of genomic selection strategies in Japanese larch.

**Supplementary Information:**

The online version contains supplementary material available at 10.1186/s12864-023-09891-4.

## Introduction

 Genetic improvement of economically and ecologically important species depends on accurate decomposition of phenotypic variation and prediction of breeding values for traits of interest. Phenotypic variation can be decomposed into genetic, environmental, and interaction variance according to quantitative genetic theory [1]. Genetic variance can be further decomposed into additive and non-additive variance (dominance, imprinting and epistasis). Traditionally, the genetic effects are estimated upon pedigree information. For instance, the additive effect can easily be obtained from a half-sib progeny test. In contrast, the analysis of non-additive variance requires the establishment of a more complex family structure (e.g., full-sib families) [2]. Typically, full-sib families are derived from crossbreeding, which is time-consuming and logistically challenging, especially in the early stages of breeding cycles, or impractical for some species in the early stages of breeding, such as many forest trees. Pedigree reconstruction is an alternative method to complete the unknown male-parent information by molecular markers and paternity analysis on half-sib families [3]. However, the expected theoretical relationships between members of half- or full-sib families may bias the estimates of variance component, breeding value, and expect genetic gain for the Mendelian sampling effect [4]. Fortunately, the use of Genomic-based Best Linear Unbiased Prediction (GBLUP) and the reduced cost of genotyping provide the opportunity not only to estimate the non-additive effect [5–7] but also to obtain more accurate genetic parameters [8–10] using the realized relationship matrix constructed by molecular markers, facilitating a better understanding of the genetic basis of key traits. Moreover, the non-additive effects could be selectively considered into genomic selection models based on their contributions.

Genomic selection (GS), introduced by Meuwissen et al. in 2001 [11], uses the developed models and genomic markers to predict genomic breeding values of selection candidates, offering the potential to significantly shorten the breeding cycle and increase gains per unit time. These advantages make GS attractive for improving the traits of animals (e.g. [12, 13]), crops (e.g. [14]), and especially long-lived forest trees (e.g. [15, 16]). Typically, non-additive effects and prediction methods are important aspects that need to be carefully considered when developing GS models. Both simulation and empirical studies have been carried out to evaluate the impact of non-additive effects on the predictive ability of the models [17–20]. Dominance improved the prediction of complex traits in plants, such as grain production and drought tolerance in maize hybrids [21], yield in sorghum [22], or growth in hybrid *Eucalyptus* [23]. Epistasis can influence the accuracy of GS for complex traits in plants. Jiang et al. [24] found that epistatic effects played a more prominent role in grain-yield heterosis in wheat using GS. Raffo et al. [25] showed that epistatic models can be useful to enhance predictions of total genetic merit for wheat grain yield. However, the inclusion of epistasis in GS models may reduce the accuracy of the models [26]. In addition, few studies have been focused on imprinting in GS [7, 27], although this non-additive effect has been shown to play a role in the expression of several phenotypes in plants [28] and animals [29]. It also should be noted that the influence of non-additive effects on GS may depend on factors such as the type of trait under consideration, the genetic architecture of the trait, and the size and structure of the population being analyzed. A number of prediction methods have been proposed for GS, such as Genomic Best Linear Unbiased Prediction (GBLUP), Ridge Regression, Bayesian-based models, and Reproducing Kernel Hilbert Spaces (RKHS) [30, 31], and the performance of these methods needed to be evaluated in a GS programme. Previous empirical studies have shown that for traits genetically regulated by a large number of genes with small effects, there is no statistically significant difference in performance between GBLUP and Bayesian-based models [32–37]. The Reproducing Kernel Hilbert Space (RKHS) model is a powerful mathematical framework used in machine learning and statistical learning theory that provides a way to represent and analyze data in a high-dimensional feature space using kernel functions [30, 31]. When non-additive effects, particularly epistasis, play a role in a trait, Reproducing Kernel Hilbert Spaces (RKHS), which combine features of non-parametric kernel regression with mixed-effect linear models [38], usually have a better predictive ability [39–41]. Therefore, it is both necessary and promising to determine the contribution of non-additive effects and then to assess the predictive ability of RKHS models using GBLUP as a benchmark for those traits that are significantly regulated by non-additive effects.

Japanese larch (*Larix kaempferi* (Lam.) Carrière) is an economically and ecologically important tree characterized by rapid juvenile growth rate and wide environmental adaptability. It is one of the most successful exotic species and is rapidly becoming the preferred conifer for lumber and pulpwood production in northern China and the southern sub-alpine region. In 1965, a 14.7 ha clonal seed orchard was established at the Dagujia Tree Farm in Liaoning Province, China (42.3–42.4°N latitude, 124.8-125.2°E longitude, with an altitude of 200–600 m). Plantations established in the northeastern China in the early twentieth century provided the clones with outstanding growing performance. For many years, these clones have been the subject of progeny testing through open-pollinated and control-pollinated crosses. The genetic material for this study came from one of the progeny tests, which was established in 1988, which consists of 94 open-pollinated (OP) and 55 control-pollinated (CP) families. Based on this population, we have investigated the additive and dominant effects, spatial patterns and competition for growth traits [42, 43], implemented pedigree reconstruction using microsatellite markers [44], and evaluated the influence of pedigree reconstruction on genetic parameter estimation [45]. In this study, the genetic research and the GS model development were carried out on a sample of 661 trees from 66 OP families. Individual trees were phenotyped for nine valuable traits related to growth traits, wood physical and chemical traits, and competition index and were genotyped using 11,333 high-quality SNPs captured by the Genotyping by Sequencing (GBS). The main objectives of this study were to (1) compare the model fitting and parameter estimates between the OP-pedigree and full-sib (FS) pedigree reconstructed in our previous study; (2) determine the contribution of additive, dominance, imprinting, and first-order epistasis using the GBLUP method and construct a GS model for each trait based on the importance of non-additive components; and (3) assess the predictive ability of the RKHS models using GBLUP as a benchmark for each trait, and select the better one for further application.

## Materials and methods

### Genetic material

The data used in this study are from a progeny trial of the Japanese larch tree improvement programme in the Temperate Breeding Zone, China. The field trial, transplanted to the field in 1988 with 2-year-old seedlings, compromises 94 open-pollinated families. The seeds of the open-pollinated families were collected from the first-generation seed orchard of Japanese larch in China, established with 152 maternal clones [44]. The field design was a randomized complete block design with 5 replications for each family. The number of trees per family varied from 5 to 15 per replications and the number of 5-tree row plot per family varied from 1 to 3. 5-tree row plots with 1–3 columns due to seedling availability and 2 × 2 m spacing. This study involved 661 randomly selected trees from 66 open-pollinated families with 9–12 trees per family.

### Phenotypic data

#### Growth

In autumn of 2005 (age 19), all trees in the trial were assessed and measured for growth traits. Tree height (H, m) was measured using a Vertex III sonic clinometer (Haglöf Sweden AB, Västernorrland, Sweden). Diameter at breast height (DBH, cm) was measured using a diameter tape. Tree stem volume (V, dm^3^) was calculated by the following equation developed for larch trees in eastern Liaoning Province (Liao Q1667-83):

1 $$V=0.0592372\times{DBH}^{2}\times {H}^{0.98098926}$$

#### Competition index

DBH data were used to calculate competition indices to characterize the competitive ability of individuals to their neighbors. The Hegyi index, proposed by Hegyi in 1974 [46], is the most classical spatial competition index used in forest research to quantify the competitive pressure of trees in forests [47] Hegyi’s competition index depends on the size and distance of neighboring trees, and was calculated as follows:

2 $$CI=\sum _{j=1}^{n}\left(DB{H}_{j}/DB{H}_{i}\right)/{D}_{ij}$$

where $i$ is the $i$th tree, $j$ is the $j$th neighbor of this tree, $1\le n\le 8$, ${D}_{ij}$ is the distance between the $i$th tree with its $j$th neighbor. The first-order neighbors were considered in this study, so ${D}_{ij}$=2 m (in row or column direction) or $2\sqrt{2}$ m (in diagonal direction).

#### Wood physical traits

##### **Pilodyn penetration**

The Pilodyn 6 J Forest penetrometer (PROCEQ, Switzerland) with a 2.0 mm diameter pin was inserted into each stem twice, without removing the bark, in the southern and northern directions at approximately 1.3 m above ground. The average Pilodyn penetration (PILODYN) was used as a surrogate for the wood density characteristic.

##### Acoustic assessment

Acoustic velocity (AV) is directly related to dynamic wood stiffness or modulus of elasticity (MOE) in conifers and has been widely used as a proxy [48–50]. The AV of each selected and sampled tree was measured using the Fakkop Microsecond Timer tool (Fakopp Enterprise, Ágfalva, Hungary) to substitute the dynamic modulus of elasticity estimate at age 32. Two probes were inserted lengthwise into the tree trunks at heights of 0.3 and 1.3 m. Two measurements taken from both the north and south sides of the trunk and averaged before analysis.

#### Wood chemical traits

##### Sample preparation

In winter 2017 (age 27), one to three 5 mm increment cores were extracted from each sampled tree at breast height $\pm$ 10 cm from bark to bark with an increment borer to meet the needs of subsequent NIR scanning. Cores without bark were air dried to constant weight (wood samples were dried to an equilibrium moisture content of 12%), ground to wood meal, filtered through a 60-mesh sieve and stored in sealed plastic bags for subsequent NIR spectra scanning.

##### Spectra collection

Near infrared (NIR) spectra were recorded using a Field Spec® spectrophotometer (ASD Inc., Boulder, CO) with wavelengths between 350 and 2500 nm at 1 nm intervals. The room temperature and humidity were kept constant. The spectrophotometer was pre-run for 30 min before each regular measurement. Three measurements of NIR spectra were taken per sample and averaged before statistical analysis. The contents of three wood chemical properties, including holo-cellulose, hemi-cellulose and lignin, were predicted using NIR-PLS models developed for *L. kaempferi* [51] and denote by HOLOCEL, HEMICEL and LIGNIN.

### Pedigree reconstruction

Because we have previously reported the results of the pedigree reconstruction [44], the material, methods and results are briefly outlined here. A total of 17 simple sequence repeat (SSR) markers were used for genotyping 647 progenies from 63 open-pollinated families and 140 existing maternal clones. 62 families were shared between the previous and the present studies. The CERVUS software (version 4.0) [52] was used to perform paternity analysis with a strict confidence level (CL) of 95% and a relaxed CL of 80% in 10,000 simulation cycles. 572 progenies were implicitly assigned to 97 paternal clones, of which 223 were at the strict CL and the rest at the relaxed CL, generating 433 full-sib families with a mean number of trees of 1.21 (SD = 0.63) per full-sib family. The limited number of trees per full-sib family may bias genetic parameter estimates [53, 54]. Therefore, the reconstructed pedigree provides preliminary estimates of the genetic parameters of various traits.

### SNP genotyping

DNA extraction was performed on fresh needles from the sample trees using a CTAB procedure modified from Doyle and Doyle [55]. To generate a high-density SNP profile for the 661 DNA extracts, we conducted a multiplexed, high-throughput Genotyping-by-Sequencing (GBS) following Elshire et al. [56]. A 48-plex GBS library containing 47 DNA samples and a negative control (no DNA) was prepared and each of the 47 DNA extracts was barcoded. Briefly, each DNA extract (500 ng) was digested with the restriction enzyme ApeKI for 2 h. Ligation products from each DNA extract were pooled and purified using the QIAquick PCR purification kit (Qiagen). The amplified 48-plex libraries were diluted and sequenced twice (single-end reads only) on the Illumina HiSeq 2000 to achieve the sequencing coverage equivalent to 24-plex. Raw DNA short-read sequences were analyzed with a pipeline, the Universal Network Enabled Analysis Kit (UNEAK), tailored to species lacking reference genome information. To reduce sequencing errors in genotyping, we set the error tolerance rate to 0.03 (to exceed the expected Illumina sequencing error rate of 0.4%). The resulting SNP table was further filtered using the minimum value of inbreeding coefficient (mnF = 0.05) and minimum minor allele frequency (mnMAF = 0.05), and SNPs that are present in less than 40% of the samples were eliminated from further analysis. Finally, a total of 11,333 SNPs was used for genotyping the individual trees (see Additional file 1: Table S1). Accuracy validation of SNP calling was performed using conventional PCR and Sanger-based sequencing (see Additional file 1: Table S2) in 30 progenies. A total of eight fragments containing 69 SNPs were randomly selected and 54 SNPs (78.3%) were validated. The mean missingness of genotypic data was 17.7%. Missing data were imputed using random imputation method implemented in the R package synbreed [57] in the R environment (version 4.0.2) [58].

### Statistical models

For each trait, three pedigree-based individual tree models (ABLUP-OP, ABLUP-FS-A and ABLUP-FS-AD) and four genomic-based individual tree models (GBLUP-A, GBLUP-AD, GBLUP-ADI, GBLUP-ADIE) were fitted.

For the pedigree-based models, ABLUP-AD was the full model, and the rest were reduced models. The form of ABLUP-AD was as follows:

3 $$y=X\beta +{Z}_{p}p+{Z}_{a}a+{Z}_{d}d+\in$$

where y is the vector of phenotypic observations of a single trait; $\beta$ is the vector of fixed effects, including a grand mean and block effects; $p$, $a$, $d$, and $\in$ are the vectors of random plot, additive, dominance, and residual effects, respectively; $X$, ${Z}_{p}$, ${Z}_{a}$, ${Z}_{d}$ and ${Z}_{e}$ are the incidence matrices for $p$, $a$, $d$, and $\in$, respectively.

Assuming that the random effects in formula (3) follow a joint distribution as:

4 $$\left[\begin{array}{c}p\\ a\\ d\\ \in\end{array}\right]\sim N\left(\left[\begin{array}{c}0\\ 0\\ 0\\ 0\end{array}\right],\left[\begin{array}{cccc}I{\sigma }_{p}^{2}& 0& 0& 0\\ 0& {A}_{A}{\sigma }_{a}^{2}& 0& 0\\ 0& 0& {A}_{D}{\sigma }_{d}^{2}& 0\\ 0& 0& 0& I{\sigma }_{\in}^{2}\end{array}\right]\right)$$

where ${A}_{A}$ and ${A}_{D}$ are pedigree-based relationship matrices for additive and dominance effects, respectively; $I$ is the identical matrix; ${\sigma }_{p}^{2}$, ${\sigma }_{a}^{2}$, ${\sigma }_{d}^{2}$, and ${\sigma }_{\in}^{2}$ are variances of plot, additive, dominance, and residual effects, respectively.

The matrices ${A}_{A}$ and ${A}_{D}$ were constructed by the kin function in the R package synbreed [57].

For the genomic-based models, GBLUP-ADIE was the full model, and the rest were reduced models. The form of GBLUP-ADIE was as follows:

5 $$y=X\beta +{Z}_{p}p+{Z}_{a}a+{Z}_{d}d+{Z}_{i}i+{Z}_{e}e+\in$$

where $i$ and $e$ are the vectors of random imprinting and additive-by-additive epistatic effects, respectively; ${Z}_{i}$ and ${Z}_{e}$ are the incidence matrices for $i$ and $e$, respectively. The other terms are as defined in formula (3).

Assuming that the random effects in formula (5) follow a joint distribution as:

6 $$\left[\begin{array}{c}p\\ a\\ d\\ i\\ e\\ \in\end{array}\right]\sim N\left(\left[\begin{array}{c}0\\ 0\\ 0\\ 0\\ 0\\ 0\end{array}\right],\left[\begin{array}{cccccc}I{\sigma }_{p}^{2}& 0& 0& 0& 0& 0\\ 0& {G}_{A}{\sigma }_{a}^{2}& 0& 0& 0& 0\\ 0& 0& {G}_{D}{\sigma }_{d}^{2}& 0& 0& 0\\ 0& 0& 0& {G}_{I}{\sigma }_{i}^{2}& 0& 0\\ 0& 0& 0& 0& {G}_{E}{\sigma }_{e}^{2}& 0\\ 0& 0& 0& 0& 0& I{\sigma }_{\in}^{2}\end{array}\right]\right)$$

where ${G}_{A}$, ${G}_{D}$, ${G}_{I}$, and ${G}_{E}$ are genomic-based relationship matrices for additive, dominance, imprinting, and additive-by-additive epistatic effects, respectively; ${\sigma }_{i}^{2}$ and ${\sigma }_{e}^{2}$ are variances of imprinting and additive-by-additive epistatic effects, respectively. The other terms are as defined in formula (4).

The genomic-based relationship matrices ${G}_{A}$, ${G}_{D}$, ${G}_{I}$, and ${G}_{E}$ were constructed from SNP data as follows [59, 60]:

7 $${G}_{A}=\frac{{M}_{A}{M}_{A}^{{\prime }}}{2\sum {p}_{j}\left(1-{p}_{j}\right)}$$

8 $${G}_{D}=\frac{{M}_{D}{M}_{D}^{{\prime }}}{\sum {\left(2{p}_{j}\left(1-{p}_{j}\right)\right)}^{2}}$$

9 $${G}_{I}=\frac{{M}_{I}{M}_{I}^{{\prime }}}{\sum {\left(2{p}_{j}\left(1-{p}_{j}\right)\right)}^{2}}$$

where ${M}_{A}$, ${M}_{D}$ and ${M}_{I}$ are $n\times p$ matrices, $n$ and $p$ are the number of individuals and SNPs, respectively. ${p}_{j}$ is the observed minor allele frequency of the *j*th SNP. The elements of ${M}_{A}$, ${M}_{D}$ and ${M}_{I}$ for the $i$th individual at the $j$th SNP are calculated as follows:

10 $${M}_{Aij}=\left\{\begin{array}{ll}0-2{p}_{j}& \left({A}_{1}{A}_{1}\right)\\ 1-2{p}_{j}& \left({A}_{1}{A}_{2} \text{and} {A}_{2}{A}_{1}\right)\\ 2-2{p}_{j}& \left({A}_{2}{A}_{2}\right)\end{array}\right.$$

11 $${M}_{Dij}=\left\{\begin{array}{ll}-2{p}_{j}^{2}\left({A}_{1}{A}_{1}\right)& \left({A}_{1}{A}_{1}\right)\\ 2{p}_{j}\left(1-{p}_{j}\right)& \left({A}_{1}{A}_{2} \text{and} {A}_{2}{A}_{1}\right)\\ -2{\left(1-{p}_{j}\right)}^{2}& \left({A}_{2}{A}_{2}\right)\end{array}\right.$$

12 $${M}_{Iij}=\left\{\begin{array}{ll}0& \left({A}_{1}{A}_{1}\right)\\ 1& \left({A}_{1}{A}_{2}\right)\\ -1& \left({A}_{2}{A}_{1}\right)\\ 0& \left({A}_{2}{A}_{2}\right)\end{array}\right.$$

The first-order epistatic relationship matrix ${G}_{AA}$ was computed using the Hadamard product (cell by cell multiplication, denoted by #). The additive-by-additive term is ${G}_{AA}={G}_{A}\#{G}_{A}$. These matrices were constructed using the sommer-R package (version 4.0) [58, 61].

For ABLUP and GBLUP model analyses, restricted maximum likelihood (REML) estimates of (co)variance components were obtained by using the average information (AI) algorithm, implemented in the sommer-R package (version 4.0) [61]. All (co)variance component estimates were positively constrained.

The coefficient of variation *CV* was calculated as follows:

13 $$CV=\sigma_P/\overline X\times100\text{\%}$$

where ${\sigma }_{P}$ and $\overline X$ are the square root of the phenotypic variance and the grand mean, respectively.

The narrow and broad sense heritability were estimated as follows:

14 $$h^2=\frac{{\sigma }_{a}^{2}}{{\sigma }_{P}^{2}}$$

15 $$H^2=\frac{{\sigma }_{g}^{2}}{{\sigma }_{P}^{2}}$$

where ${\sigma }_{a}^{2}$ is the estimated additive genetic variance; ${\sigma }_{g}^{2}$ is the sum of all the genetic effect variances; ${\sigma }_{P}^{2}$, the phenotypic variance, is the sum of all the variances of the random effects. Both ${\sigma }_{g}^{2}$ and ${\sigma }_{P}^{2}$ were different in different models. In model (5), for example, ${\sigma }_{g}^{2}={\sigma }_{a}^{2}+{\sigma }_{d}^{2}+{\sigma }_{i}^{2}+{\sigma }_{e}^{2}$, ${\sigma }_{P}^{2}={\sigma }_{p}^{2}+{\sigma }_{g}^{2}+{\sigma }_{\in}^{2}$. In the remaining models, the term for genetic effect variance was specifically omitted from the formular according to the form of the model.

The Akaike Information Criterion (AIC, [62]) and Schwarz­Bayesian Information Criterion (BIC, [63]) were used to compare the relative quality of the goodness-of-fit of the different models with a threshold of 2. $AIC=-2logL+2\rho$, $BIC=-2logL+2log\left(n\right)\rho$, where $logL$ is the REML log-likelihood, $\rho$ is the number of estimated parameters, *n* is the number of observations. A smaller AIC or BIC value indicates a better quality of fit.

Based on the estimates of the (co)variance components and the information criteria values, we omitted the genetic terms that provide a negligible contribution to the phenotypic variance and re-fitted the GBLUP models and RKHS models, i.e. the optimal model for each trait.

RKHS models assume that the random effects in formula (5) follow a joint distribution as:

16 $$\left[\begin{array}{c}p\\ a\\ d\\ i\\ e\\ \in\end{array}\right]\sim N\left(\left[\begin{array}{c}0\\ 0\\ 0\\ 0\\ 0\\ 0\end{array}\right],\left[\begin{array}{cccccc}I{\sigma }_{p}^{2}& 0& 0& 0& 0& 0\\ 0& {K}_{A}{\sigma }_{a}^{2}& 0& 0& 0& 0\\ 0& 0& {K}_{D}{\sigma }_{d}^{2}& 0& 0& 0\\ 0& 0& 0& {K}_{I}{\sigma }_{i}^{2}& 0& 0\\ 0& 0& 0& 0& {K}_{E}{\sigma }_{e}^{2}& 0\\ 0& 0& 0& 0& 0& I{\sigma }_{\in}^{2}\end{array}\right]\right)$$

each of ${K}_{A}$, ${K}_{D}$, ${K}_{I}$, and ${K}_{E}$ depends on a reproducing kernel function with a smoothing semi-parameter *h*, which controls how quickly the prior covariance function declines with increasing genomic distance between genotypes and can be interpreted as a correlation matrix [64]. The other terms are as defined in formula (6). The RKHS models were implemented using the BGLR function from the BGLR package in R [65]. The Gibbs chain length was 20,000 iterations with the first 2000 iterations discarded as burn-in and a thinning interval set to 100.

A 10-fold cross-validation scheme with 10 replicates was used to assess accuracy and predictive ability (PA). The PA of the models was evaluated as the Pearson correlation coefficient between the predicted breeding values of the validation trees and the phenotypes adjusted for block effects and was compared using GBLUP-A as a benchmark.

## Results

### Phenotypic variation

Descriptive statistics for each trait are presented in Table 1. The *CV* of wood chemical traits were generally lower than those of growth and wood physical traits. Estimates of *CV* for HOLOCEL and LIGNIN were 4.14% and 7.44%. HEMICEL had the highest *CV* among the wood chemical attributes (11.12%). In wood physical properties, *CV* for PILODYN was 11.42%, higher than that for AV (*CV* = 9.24%). Estimates of *CV* for H, DBH and V were 11.37%, 14.55% and 34.97%, respectively. The *CV* of CI was the highest of all traits, at 44%.

**Table 1:** Descriptive statistics for the analyzed traits

Traits	Mean	SD	Min	Max	*CV*/%
H/m	17.72	2.02	10.50	24.00	11.37
DBH/cm	16.66	2.42	9.10	31.10	14.55
V/dm^3^	195.68	68.43	43.16	621.68	34.97
CI	1.08	0.47	0.22	3.23	43.49
PILODYN	20.20	2.31	13.00	26.00	11.42
AV	232.67	21.50	186.00	399.00	9.24
HOLOCEL/%	70.81	2.93	63.53	82.04	4.14
HEMICEL/%	29.94	3.33	17.79	39.42	11.12
LIGNIN/%	27.35	2.04	21.16	32.98	7.44

Note: *H *Tree height, *DBH *Diameter at breast height, *V *Stem volume, *CI *Competition index, *PILODYN *Pilodyn penetration, *AV *Acoustic velocity, *HOLOCEL *Holo-cellulose, *HEMICEL *Hemi-cellulose, *LIGNIN *Lignin, *Max* Maximum value, *Min *Minimum value, *SD *Standard deviation, *CV* Coefficient of variation

### Model comparison

AIC and BIC values obtained from the 7 models are presented in Table 2. The AIC values were generally in agreement with the BIC values of the same model. The comparison of ABLUP-OP and ABLUP-FS showed that ABLUP-OP outperformed ABLUP-FS for growth and wood chemical properties except for DBH. ABLUP-OP and ABLUP-FS showed no differences for CI and wood physical properties. The comparison of ABLUP and GBLUP showed that ABLUP outperformed GBLUP for wood chemical properties, while GBLUP performed better than ABLUP for CI and wood physical properties; the patterns for growth were irregular. The comparison of GBLUP showed that GBLUP-A and other models showed no differences for wood chemical properties; GBLUP-ADIE was generally the best for growth, CI and AV. These results showed that (1) the performance of ABLUP was generally better than GBLUP-A for wood chemical traits, (2) epistasis played a more important role in model fitting for growth, CI and AV, and (3) dominance and imprinting contributed slightly to model fitting for almost all traits.

**Table 2:** AIC and BIC values of the 7 models (3 ABLUP and 4 GBLUP models) for the analyzed traits

Traits	Information criterion	ABLUP-OP	ABLUP-FS-A	ABLUP-FS-AD	GBLUP-A	GBLUP-AD	GBLUP-ADI	GBLUP-ADIE
H	AIC	380.95	383.44	383.38	380.22	380.15	380.15	377.81
H	BIC	434.85	437.35	437.29	434.13	434.06	434.06	431.72
DBH	AIC	540.30	542.43	541.89	545.82	544.99	544.99	543.83
DBH	BIC	594.20	596.34	595.80	599.72	598.90	598.90	597.73
V	AIC	507.72	510.19	510.17	510.42	509.18	509.18	507.23
V	BIC	501.79	564.09	564.07	501.09	500.12	500.12	492.84
CI	AIC	587.40	587.98	587.93	583.46	583.46	580.73	578.08
CI	BIC	640.94	641.52	641.47	637.00	637.00	634.27	631.62
PILODYN	AIC	529.82	528.30	528.30	514.51	514.42	510.97	NA
PILODYN	BIC	559.86	558.34	558.34	544.55	544.46	541.01	NA
AV	AIC	522.91	524.02	523.30	518.65	516.46	516.46	502.68
AV	BIC	576.78	577.89	577.17	572.52	570.33	570.33	556.55
HOLOCEL	AIC	366.71	440.06	438.85	463.51	463.51	462.90	462.90
HOLOCEL	BIC	414.26	487.60	486.40	511.06	511.06	510.44	510.44
HEMICEL	AIC	390.52	459.08	NA	469.59	469.59	469.59	469.59
HEMICEL	BIC	438.07	506.62	NA	517.14	517.14	517.14	517.14
LIGNIN	AIC	363.73	423.96	423.96	462.94	462.56	462.56	NA
LIGNIN	BIC	411.27	471.51	471.51	510.49	510.10	510.10	NA

Note: See Table 1 for full description of traits. AIC: Akaike Information Criterion; BIC: Schwarz­Bayesian information criterion; ABLUP-OP: the OP pedigree-based individual tree model (using the additive relationship matrix ${A}_{A}$ estimated from the OP pedigree with known maternity only); ABLUP-FS-A: the FS pedigree-based individual tree model (using the additive relationship matrix ${A}_{A}$ matrix estimated from the FS pedigree reconstructed using paternity assignment analysis); ABLUP-FS-AD: the FS pedigree-based individual tree model (using the additive and dominance matrices ${A}_{A}$ and ${A}_{D}$ estimated from the FS pedigree reconstructed using paternity assignment analysis); GBLUP-A: the genomic selection model (using the realized additive genomic relationship matrix ${G}_{A}$ estimated from SNPs); GBLUP-AD: the genomic selection model (using the realized additive and dominance genomic relationship matrices ${G}_{A}$ and ${G}_{D}$ estimated from SNPs); GBLUP-ADI: the genomic selection model (using the realized additive, dominance and imprinting genomic relationship matrices ${G}_{A}$, ${G}_{D}$ and ${G}_{I}$ estimated from SNPs); GBLUP-ADIE: the genomic selection model (using the realized additive, dominance, imprinting and additive-by-additive epistatic genomic relationship matrices ${G}_{A}$, ${G}_{D}$, ${G}_{I}$ and ${G}_{E}$ estimated from SNPs); NA: not available due to due to failure of the model to reach convergence

### Genetic variance components

**Fig. 1:**
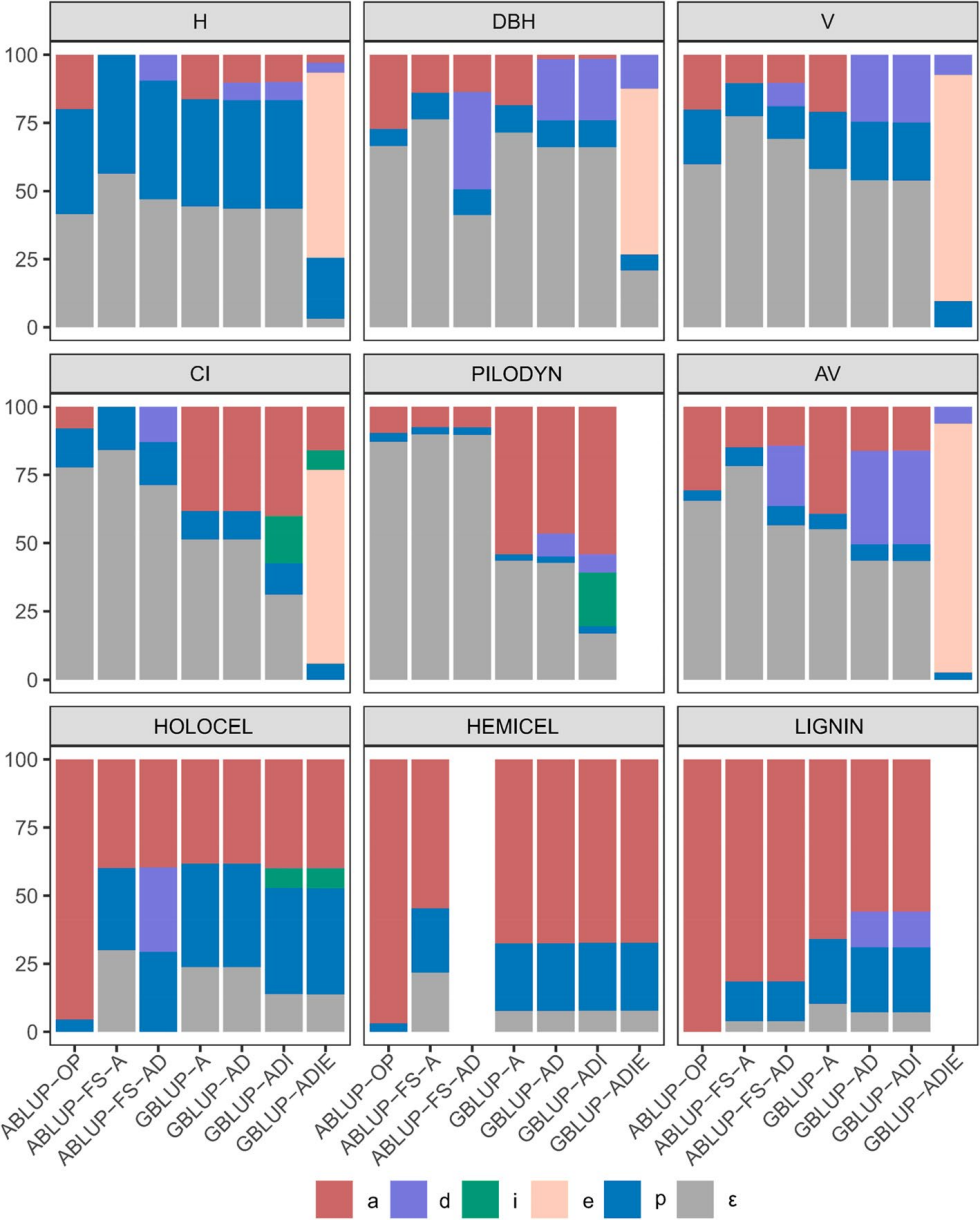
The proportion of variance for analyzed traits from the 7 genetic models fitted. See Table 1 for full description of traits. See Table 2 for full description of models. $a$, $d$, $i$, $e$, $p$, and $\in$ are the variance proportion of additive, dominance, imprinting, additive-by-additive epistatic effects, plot, and residual effects, respectively

The proportions of variance components (additive ${\sigma }_{a}^{2}$, dominance ${\sigma }_{d}^{2}$, imprinting ${\sigma }_{i}^{2}$, epistatic ${\sigma }_{e}^{2}$, plot ${\sigma }_{p}^{2}$ and residual effects ${\sigma }_{\in}^{2}$) obtained from the 7 models are presented in Fig. 1.

The comparison of ABLUP-OP and ABLUP-FS showed that the proportions of ${\sigma }_{a}^{2}$ estimated from ABLUP-OP (7.88-100%) were larger than those from ABLUP-FS (0-81.41%) for all traits, indicating that they were estimated upwards from ABLUP-OP; ${\sigma }_{d}^{2}$ was observed in ABLUP-FS-AD (8.48-35.61%) for growth and AV, as well as in GBLUP-AD (6.48-34.30%). The comparison of ABLUP-OP and GBLUP-A showed that the proportions of ${\sigma }_{a}^{2}$ estimated from ABLUP-OP (95.48-100%) were larger than those from GBLUP-A (38.20-67.46%) for wood chemical properties; in contrast, for CI and wood physical properties, ${\sigma }_{a}^{2}$ contributed more of the variance from GBLUP-A (38.17-54.11%) than ABLUP-OP (7.88-30.67%).

The comparison of GBLUP showed that additive effect is the main source of variation for wood chemical properties (38.20-67.46%). Except for growth and AV, ${\sigma }_{d}^{2}$ had contribution to CI and HOLOCEL in ABLUP-FS-AD and to PILODYN and LIGNIN in GBLUP-AD. Dominance variances were generally separated from the residual variances in ABLUP-FS-AD. However, in GBLUP-AD, the dominance variances were more from additive genetic variances and a small part of residual variance: up to half of ${\sigma }_{a}^{2}$ was reduced for AV, and almost all of ${\sigma }_{a}^{2}$ was reduced to near zero for DBH, V and PILODYN. These results indicated that ${\sigma }_{a}^{2}$ estimated from GBLUP-A may confounded with the ${\sigma }_{d}^{2}$, and that including dominance effect could improve the explanation of residual variance for those traits that are considerable genetically controlled by dominance effect. The imprinting effects had contributions to CI, PILODYN and HOLOCEL, with proportions estimated by GBLUP-ADI of 17.33%, 19.71% and 7.08%, respectively. The imprinting effect variances were dissected from the residual variances. For growth, CI and AV, when epistasis was considered, a surprisingly significant proportion of the variance was explained by this effect (50.76-91.26%), which was decomposed from other sources of variation, particularly the residual variance. These results were generally consistent with the AIC values. Therefore, the traits could be classified into two types: for type I, including wood chemical properties and PILODYN, additive effect is the main source of variation; for type II, including growth, CI and AV, epistasis plays a significant role.

### Heritability estimates

**Fig. 2:**
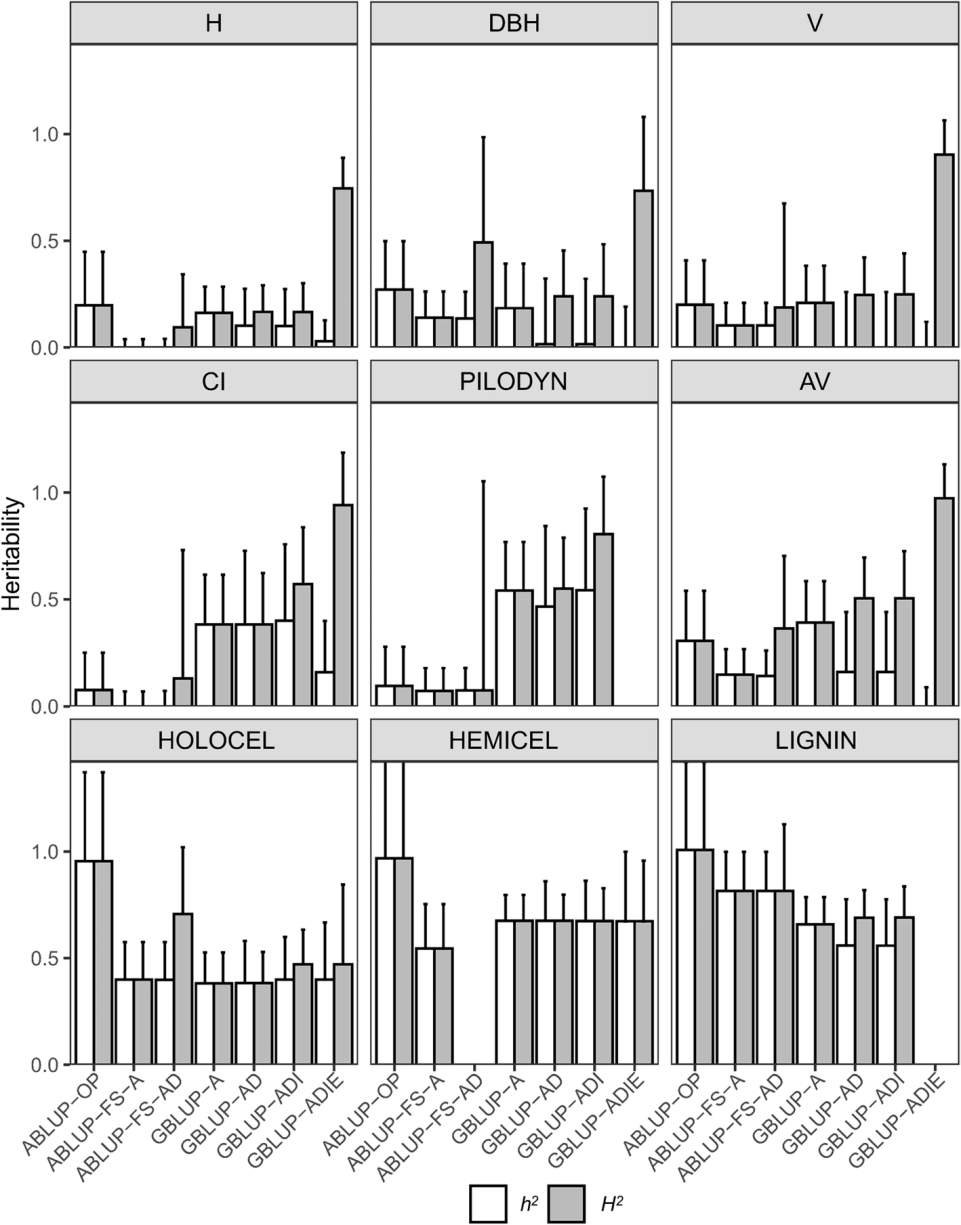
The narrow and broad sense heritability for each trait from the 7 genetic models fitted. $h^2$ and ${H}^{2}$ are the narrow and broad sense heritability, respectively. Error bars above each bar indicate standard errors (SE). See Table 1 for full description of traits. See Table 2 for full description of models

Narrow and broad sense heritability ($h^2$ and $H^2$) exhibited similar trends as additive and total genetic variance respectively from different models and are shown in Fig. 2. $h^2$ estimates from ABLUP-FS were lower than those from ABLUP-OP for all traits. For growth traits, $h^2$ estimates from ABLUP-OP indicated that these traits were moderately controlled by additive effects, ranging from 0.20 to 0.27, which were slightly higher than those from GBLUP-A (0.16–0.20). For wood physical properties and CI, $h^2$ estimates from GBLUP-A, 0.38–0.54, were much higher than those from ABLUP-OP (0.08–0.31).

As the number of genetic terms increased, the ${\sigma }_{a}^{2}$ were partitioned into other components, thus $h^2$ were gradually reduced to zero for traits such as DBH. On the other hand, the $H^2$ were increased from 0.17 to around 0.80, due to the inflating non-additive components, which are mainly epistatic effects.

The wood chemical properties showed the highest $h^2$ estimates in this study. $h^2$ estimates from ABLUP-OP were almost one seemingly unrealistic. The $h^2$ and $H^2$ estimates did not show much fluctuation when more genetic effects were included in the GBLUP models, ranging from 0.38 (HOLOCEL) to 0.67 (HEMICEL). The $h^2$ and $H^2$ estimates from ABLUP-OP and ABLUP-FS had higher standard errors than those from GBLUP-A in most cases, indicating that more accurate $h^2$ and $H^2$ estimates could be obtained using the GBLUP method.

#### Predictive ability

**Fig. 3:**
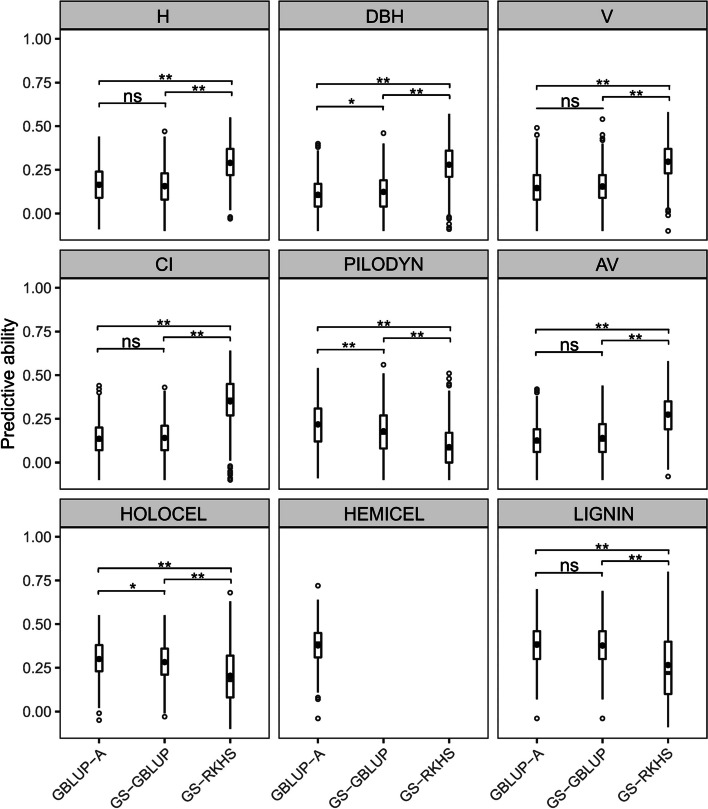
Distribution of predictive abilities for each trait. Each panel contains box plots showing the distribution of predictive abilities from the base models (GBLUP-A), and the optimal models (without genetic terms that provide a negligible contribution to the phenotypic variance) considering non-additive effects fitted by the GBLUP (GS-GBLUP) and RKHS (GS-RKHS) approaches. ns represents non-significance; * and ** indicate significant at *p* = 0.05 and *p* = 0.01. Hollow circles represent outliers. The black solid points and bars represent means and medians. See Table 1 for full description of traits

The PAs from the three models for each trait are presented in Fig. 3 using box plots combining the results of the hypothesis tests.

No significant difference was found between the PAs of GBLUP-A and the corresponding GBLUP models considering non-additive effects (GS-GBLUP) for all the traits except for DBH and HOLOCEL. For growth-related traits, the PAs of GS-RKHS were significantly higher than those of GBLUP-A and GS-GBLUP (*p* < 0.01). The PAs of GS-RKHS for H, DBH, and V were ~ 0.29, 89.30-148.26% higher than those of GS-GBLUP, which were 0.15, 0.10, and 0.15, respectively. For CI, the PA of GS-RKHS was 0.35, 149.08 times higher than that of GS-GBLUP. For PILODYN, a wood physical trait, the PA of GS-RKHS was 0.05, significantly lower than 0.18 of GS-GBLUP and 0.22 of GBLUP-A. For AV, the pattern of PAs of the GS models was similar to that of the growth-related traits. The PA of GS-RKHS was 0.27, significantly higher than the 0.13 of GS-GBLUP. For wood chemical traits, GS-GBLUP and GS-RKHS were not successfully fitted for HEMICEL. The PAs of GS-RKHS were 0.20 and 0.26 for HOLOCEL and LIGNIN respectively, significantly lower than those of GS-GBLUP and GBLUP-A (0.30 and 0.38). There was no significant difference between the last two models. The PA of GBLUP-A for HEMICEL was 0.38.

## Discussion

Unraveling the genetic control of economically and adaptively important traits in forest tree species will help meet the growing global demand for high quality wood and the increasing challenges posed by changing climates and environments. OP progeny testing is the most efficient and widely used approach to genetic analysis and screening of large numbers of individuals in terms of low cost and less time. However, under this family structure, only the additive genetic variance component can be obtained, the estimate of which is somewhat biased due to the common violation of the true half-sib assumption [45, 66]. The considerable available DNA-marker information and GS models provided us with an unprecedented opportunity to understand the genetic control of key traits of Japanese larch, which is extremely important for decision making of breeding strategy and facilitating a better exploitation of the available inherent variation for these traits in breeding programmes. This study is the first comprehensive assessment of contribution of the additive and non-additive effects on growth, wood and competitive traits of Japanese larch using a realized-genetic based model that has been proven effective in animal and plant breeding (e.g. [67, 68]).

### Genetic control of growth, wood and competitive traits

For forest tree species used for pulp production, much attention has been paid by breeders to wood chemical properties such as cellulose and lignin content, which can be readily predicted by NIR-based calibrations. In this study, cellulose and lignin content and Pilodyn penetration are mainly controlled by additive effects in both ABLUP-OP ($h^2$=0.95-1.00) and GBLUP-A ($h^2$=0.38–0.67), which have been reported in various forest tree species in both conventional and GS studies [36, 69–73]. Therefore, it is promising to achieve significant genetic gain through GS-based recurrent selection. Meanwhile, the minimal contribution of non-additive effects on these traits also demonstrated the efficacy of the previous studies that did not consider non-additive effects. Additionally, the moderate to high narrow-sense heritability indicates that the discovery of the genes underlying the biosynthesis of these traits is prospective using genome-wide association mapping studies (GWAS). In Japanese larch, 77 SNPs were significantly associated with cellulose and lignin content and were located in 54 genes (unpublished data). In *Populus tomentosa* Carrière, Li et al. [74] reported that selection of specific SNPs in functional genes could regulate the cellulose and lignin content. In *Picea abies* L. Karst, Chen et al. [75] detected four SNPs associated with Pilodyn penetration. Alternatively, the genetic gain could be increased by manipulating the genetic elements involved using molecular biology approaches.

For growth, AV and CI, additive-by-additive epistatic effects play more important role (50.76-91.26%, Fig. 1). For AV, the measurement of acoustic velocity, which represents the mechanical stiffness of wood, was moderately controlled by the additive effect from the model ABLUP-OP ($h^2$=0.31) and GBLUP-A ($h^2$=0.39) considering only the additive effect, which is consistent with other tree species reports [37, 76, 77]. However, when the epistatic effect was accounted for, almost all the phenotypic variation was explained by the epistatic and dominance effects, 91.26% and 6.08% respectively. No similar results have not been found. Epistasis showed no effect on wood stiffness in Scots pine (*Pinus sylvestris* L.) with 695 progeny trees from 184 full-sib families [9] and in control-pollinated Norway spruce (*Picea abies* (L.) Karst.) with 1370 progeny trees from 128 full-sib families [78]. Further research is required to identify the specific reasons for this result in Japanese larch. The significant contribution of epistatic effects was also found for growth and competitive traits and has also been reported in other tree species. Tree height was controlled by a significant additive-by-additive epistatic effect in an open-pollinated white spruce (*Picea glauca* (Moench) Voss) trial of 214 families [79]. In contrast, the epistatic effect could be ignored for tree height Norway spruce trials [78] and in an open-pollinated interior spruce (*Picea glauca* x *engelmannii*) test of 25 families [19]. Furthermore, an opposite contribution by epistatic effect was shown for wood density in spruce, Scots pine and hybrid *Eucalyptus* [9, 23, 79]. These inconsistent results may hinder their implementation in breeding programs.

The dominance variance was mainly absorbed by the additive variance and a small amount by the residual variance. The imprinting variance was fully extracted from the residual variance. Furthermore, the proportions of these two effects were reduced when epistasis was taken into account due to its strong capacity to absorb variance from other components [19, 80]. The dominance effect has a contribution for AV and LIGNIN (6.08% and 13.02% respectively). Dominance contributed 9.8–18.1% of phenotypic variation for tree height in Norway spruce [78] and 16.4% and 5.7% of phenotypic variation for LIGNIN and cellulose content in loblolly pine [81]. In contrast, Lenz et al. [37] reported that the dominance effect was not significant for all traits including weevil resistance, growth, and wood quality traits in Norway spruce and did not improve the model fit.

As in traditional pedigree-based genetic analysis, the population-specific genetic analysis still shown inconsistent contributions of additive and non-additive effects for the same traits in most cases in the GS period [23, 78, 82]. For those traits that are primarily controlled by non-additive effects, short-term genetic gains could be captured through vegetative propagation of individuals with high genetic values by rooted cuttings or grafts. Although somatic embryogenesis is a more efficient method for conifers, its practical application in Japanese larch is not yet well developed. For long-term genetic gain, individuals with additional genetic variation and superior characteristics should be introduced into this breeding population. Alternatively, the systematic introduction of germplasm from other larch species into the current breeding population through hybridization is a promising approach.

### RKHS with epistatic genetic effects improves predictive ability

Maximizing the predictive ability (PA) of predictive models is one of the main goals in GS, and PA has been influenced by several factors. Recent studies have shown that the PA can be improved when additive and non-additive effects are considered simultaneously in a predictive model [17–20]. In the present study, the influence of statistical model and non-additive effects on the PAs of predictive models was evaluated. The results showed that using the GBLUP approach, non-additive effects have no significant contribution to the improvement of PA for all the traits (Fig. 3), suggesting that GBLUP as a linear model may not capture complex patterns like additive-by-additive epistatic effects. In contrast, the PAs from RKHS models were significantly higher than those from GBLUP models considering additive effects only (GBLUP-A), and accounting for additive and non-additive effects simultaneously (GS-GBLUP) for those traits controlled by epistatic effects. These results showed that the main advantage of the RKHS is on its superiority in capturing epistatic effects [64, 83]. However, for traits not significantly controlled by epistatic effects but by other non-additive components, holo-cellulose and lignin content, the PAs from RKHS models were remarkably lower than those from GBLUP models, which could be attributed to the inappropriate definition of a kernel [64]. Similarly, Tan et al. [84] reported that RKHS was the worst performing method for pulp yield in two *Eucalyptus* species and their F_1_ hybrids. Therefore, for these types of traits, GBLUP models are more suitable for predicting GEBVs, in addition to their speed of fast computation. Most of the previous empirical studies showed that there was a slight difference in prediction performance for quantitative traits between GBLUP and RKHS models [32, 84–86], which could be caused by the negligible contribution of epistasis on the traits analyzed or by not accounting for epistasis in the models.

### Some issues affecting the efficiency of genomic prediction

Sample size is primarily considered in genetic research for its critical role in statistical power [87, 88]. The sample size in this study, consisting of 661 trees from 66 OP families (~ 10 individuals/family), can be considered relatively small for generating highly reliable estimates of genetic effects and genomic predictions, particularly for traits with low narrow-sense heritability (Figs. 2 and 3). In addition, the sample size is not sufficient to obtain an optimal number of individuals per full-sib family after pedigree reconstruction to precisely estimate genetic parameters for low heritability traits such as height (Fig. 2). Therefore, the reconstructed pedigree provides preliminary estimates of the genetic parameters of various traits. It is expected that the statistical power would further improve with a larger sample size [37]. However, we would like to emphasize that practical constraints, such as limited financial resources, often impose limitations on sample sizes in genetic research involving molecular genotyping. Achieving a balance between sample size and available budget is a challenge faced by many breeders [87].

Accuracy of phenotypes has significant impact on the efficiency of genomic prediction. Various non-destructive evaluation methods have been used to assess wood properties in standing trees [89]. We used Pilodyn penetration, acoustic velocity and spectroscopy techniques to proxy the wood physical and chemical properties. The Pilodyn penetration may not be a good surrogate for wood density in tree species with very high variation between earlywood and latewood densities within a growth ring, such as radiata pine (*Pinus radiata* D. Don) and *Larix kaempferi*, as the pin penetration may be affected by the number of latewood bands encountered [90]. There was also a relatively large measurement error in Pilodyn penetration, which could be caused by differences in bark thickness between trees of *Larix kaempferi*. In contrast, the Resistograph can overcome some of the limitations of Pilodyn and can serve as a more efficient alternative tool for evaluating wood density in standing trees [91] and will be used in our future investigations in *Larix kaempferi* to collect more accurate data.

## Conclusions

Genomic-based models (GBLUP) provide a more comprehensive and accurate estimate of the contribution of the additive and non-additive effects than pedigree-based models (ABLUP). In the GBLUP model with all non-additive effects, the traits considered in this study could be divided into two types based on different quantitative genetic architectures: type I, additive controlling traits, including wood chemical traits and Pilodyn penetration; type II, epistatic controlling traits, including growth traits, competitive ability and acoustic velocity. Dominance and imprinting showed low contributions to the phenotypic variance of the traits. The GBLUP and RKHS methods were preferred in terms of predictive ability for type I and type II traits, respectively.

### Supplementary Information

**Additional file 1: Table S1.** Genotyping data of 11,333 SNPs. **Table S2.** Validation of SNP calling accuracy by Sanger sequencing

## Data Availability

The raw DNA short-read sequences generated in this paper are not publicly available due to the large file size (~ 8 TB) but are available from the corresponding author on reasonable request. The original genotype and phenotype data and results were included in the article and its additional files.
